# Community perspectives on the extent to which transactional sex is viewed as sexual exploitation in Central Uganda

**DOI:** 10.1186/s12914-020-00228-w

**Published:** 2020-05-01

**Authors:** N. Kyegombe, R. Meiksin, S. Namakula, J. Mulindwa, R. Muhumuza, J. Wamoyi, L. Heise, A. M. Buller

**Affiliations:** 1 Department of Global Health and Development, London School of Hygiene and Tropical Medicine, 15-17 Tavistock Place, London, WC1H 9SH UK; 2 Department of Public Health, Environments and Society, London School of Hygiene and Tropical Medicine, 15-17 Tavistock Place, London, WC1H 9SH UK; 3 Independent researcher, Kampala, Uganda; 4 MRC/UVRI & LSHTM Uganda Research Unit, P.O Box 49, Plot 51-59 Nakiwogo Road, Entebbe, Uganda; 5 Department of Sexual and Reproductive Health, National Institute for Medical Research, P.O Box 1462, Mwanza, Tanzania; 6 Department of Population, Family and Reproductive Health, Johns Hopkins Bloomberg School of Public Health and JHU School of Nursing, 615 N. Wolfe Street, Baltimore, MD 21205 USA

**Keywords:** Uganda, Transactional sex, Sexual exploitation, Adolescent girls and young women

## Abstract

**Background:**

Definitions of child sexual exploitation vary. Sexual exploitation violates children’s rights and exposes them to mental and physical harm. There exist differences in views of behaviour that is considered exploitative, including transactional sex. This paper explores community perspectives on the extent to which transactional sex is considered exploitative.

**Methods:**

In 2014, we conducted 19 focus group discussions and 44 in-depth interviews with young people and adults in two communities in Uganda. Participants were presented with vignettes describing sexual encounters between adolescent girls and young women and men to explore under what conditions participants considered the scenario to be exploitative and why. Interviews were conducted in Luganda using a semi-structured tool, audio recorded and transcribed verbatim. Analysis was thematic and complemented by constant comparison and deviant case analysis techniques.

**Results:**

Definitions by multilateral, bilateral, and non-governmental organisations of the sexual exploitation of children shared similarities with community conceptualisations of wrong or unfair sex. Although in community conceptualisations there was no consensus on what constituted sexual exploitation, transactional sex was condemned to the extent to which it involved sex with a minor or misled a naïve or immature girl; involved lack of consent, particularly in relationships characterised by power differentials; or worsened the pre-existing status of the girl. Also relevant was the extent to which a man’s intentions were considered inappropriate; the adolescent girl or young woman was considered vulnerable; and the adolescent girl or young woman was considered responsible for ‘her situation’.

**Conclusions:**

Existing social norms that condemn sex with a minor or sex that involves deception, sexual coercion or misleading an immature girl, present opportunities to mobilise communities to protect adolescent girls and young women at risk. Any intervention must, however, be designed with full cognisance of the social and structural drivers that underlie transactional sex and limit adolescent girls’ and young women’s opportunities to provide for themselves without recourse to sexual relationships with men. Interventions must also be designed to recognise that girls in transactional sex relationships may not consider themselves as exploited, thus requiring engagement with them based on their own concerns, aspirations, and expectations.

## Background

Definitions of sexual exploitation of children in laws and conventions, policy documents, and research reports vary considerably [1]. Sexual exploitation of children is a form of child abuse that the World Health Organization defines as “the involvement of a child in sexual activity that he or she does not fully comprehend, is unable to give informed consent to, for which the child is not developmentally prepared, or else that violates the laws or social taboos of society” pg10 [2]. Melrose and Pearce further note that the “sexual exploitation of children and young people under 18 involves exploitative situations, contexts and relationships where young people (or a third person or persons) receive ‘something’ (e.g. food, accommodation, drugs, alcohol, cigarettes, affection, gifts, money) as a result of them performing, and/or another, or others performing on them, sexual activities” pg2 [3]. Moreover, the Council of Europe Convention designates sexual exploitation of children to cover situations where a child or other person is given or promised money or other forms of remuneration, payment or consideration, in return for the child engaging in sexual activity, even if the payment/remuneration is not made [4].

Sexual exploitation of children is also evidenced when sexual acts are exchanged for goods and services such as housing, food, clothing, drugs or alcohol, protection, better grades in school, or even emotional attention [1, 5]. In all cases, those exploiting the child or young person are able to do so based on power differentials derived from age, gender, intellect, physical strength and/or command of resources. Sexual exploitation of children does not always involve direct touching, can occur in any setting, and only sometimes includes force or violence. Indeed, sexual exploitation of children often involves coercion, deception, trickery, entrapment, emotional manipulation or grooming [1]. Furthermore sexually exploitative relationships (and other forms of non-sexual exploitation) can be voluntary, non-coerced, and even mutually beneficial and yet may be subject to the moral criticism that they are exploitative [6, 7]. Typically, these definitions of sexual exploitation have been defined by multilateral, bilateral and non-governmental organisations and are used to guide their work in many and varied populations and contexts around the world. They represent etic, or ‘outsider’, definitions of sexual exploitation which may not necessarily reflect the emic, or ‘insider’, views and conceptualisations of sexual exploitation in any particular community [8].

While there is some variability in etic definitions of sexual exploitation, transactional sex involving somone under the age of 18 would fall squarely into the category of child sexual exploitation when appraised against these definitions of sexual exploitation. Transactional sex is distinct from sex work, [9–15] and in Uganda, and other low- and- middle-income settings, is perpetuated by a number of social and structural drivers [9, 16–20]. Known in the Central Region of Uganda as *okwegatta okwa ‘mpa-nkuwe’ (give me, I give you sex),* transactional sex is defined as *non-marital, non-commercial sexual encounters or relationships primarily motivated by the implicit assumption that sex will be exchanged for material benefit or status* [15]*.* Young people’s engagement in transactional sex relationships has been documented in both urban and rural Uganda, [21–26] with young people reporting entering these relationships for a variety of reasons including to obtain financial or material support; in response to emotions, desires and feelings; or as a result of implicit or explicit pressure to access consumer products or achieve social status [18, 21, 23, 25, 27, 28]. Some adolescent girls and young women also strategically engage in sexual relationships with older men, often known as ‘sugar daddies,’ as a means of securing resources [18, 21, 24].

While child sexual exploitation specifically refers to someone under the age of 18, transactional sex, although a behaviour of public health importance for adolescent girls and young women, is not bounded by age. Efforts to address transactional sex often stem from epidemiological evidence of disproportionately high HIV infection rates amongst adolescent girls and young women 15–24 years of age [17, 29, 30]. With evidence of a clear association [20, 30–33], transactional sex is argued to be a potential driver of adolescent girls’ and young women’s disproportionate risk of HIV infection [16, 17, 19, 22, 30, 34–36]. Transactional sex has also been linked to sexually transmitted diseases, reproductive and sexual health risks (including unwanted pregnancy), sexual coercion, and violence [21–23, 25, 28].

Social science perspectives vary on how transactional sex is thought to be motivated and the extent to which it is viewed as exploitative. In their 2016 review and synthesis of literature on transactional sex, Stoebenau et al. found that in early studies of transactional sex, adolescent girls and young women are often characterised as ‘vulnerable victims’, who, as a result of gendered economic and social marginalisation, are forced to exchange sex for money, food, or other material support. Here, women’s economic dependence on men is emphasised, as are structural barriers to women’s ability to earn an income sufficient to meet their needs, especially in increasingly monetised economies. Under this characterisation of transactional sex, adolescent girls and young women are seen as powerless in heterosexual relationships in which they have been coerced, exploited or abused. Present in the literature, however, is the recognition that transactional sex is not confined to the exchange of sex for basic needs. Indeed, more contemporary evidence of adolescent girls’ and young women’s agency in relationships highlights instances where transactional sex appears to be motivated by relative deprivation and the pursuit of social status. This is often fuelled by economic processes of globalisation and a perceived imperative to access consumer products as symbols of upward mobility and modernity [10, 12, 15]. This characterisation of transactional sex also recognises adolescent girls’ and young women’s agency and ability to extract resources from men by using their ‘erotic power’ [37] to capitalise on traditional gendered assumptions in relationships. In many studies, such motivations are at times condemned as morally reprehensible by community members, with adolescent girls and young women who exchange sex for aspirational reasons often shamed and blamed by others [15, 17]. It is also important to note that transactional sex may also occur in emotionally intimate relationships where resource exchange adheres to social expectations of the gendered flow of resources from men to women, thus conforming to gender beliefs of men as providers of material support and women as providers of domestic and reproductive labour [15].

Recognising that adolescent girls and young women may participate in transactional sex relationships for different reasons is important for exploring people’s perceptions on the extent to which it is considered exploitative, and in turn, the scope to address harmful or exploitative aspects of the behaviour.

### Feminist theoretical framework

Drawing from data from Central Uganda, as part of Learning Initiative on Norms Exploitation and Abuse (LINEA), the aim of this paper is to explore the extent to which local people consider transactional sex to be exploitative and the degree to which this emic conceptualisation of exploitation aligns with the etic definitions outlined in this introduction. This has important implications on how effectively interventions that take an etic perspective on sexual exploitation engage with young people and communities in which these perspectives may not necessarily resonate. We focus on transactional sex relationships involving adolescent girls and young women but acknowledge that boys and young men may also be involved in such relationships. This work is guided by a feminist theoretical framework which provides a useful lens for exploring transactional sex against the backdrop of rigid gender and social norms prevalent in the patriarchal society in which this study was conducted. We explore themes including gender, age and power inequalities, the sexual objectification of adolescent girls and young women, and economic and structural inequalities that impact adolescent girls and young women’s ability to meet their needs [38–40].

## Methods

### Study context

The study was conducted in two sites in Central Uganda. The urban site, in Kampala District, is a very low-income community 5 km from the central business district. Informal sector self-employment dominates and poverty is prevalent. The rural site is located in the Masaka District, approximately 130 km southwest of Kampala. Many rely on self-employment in the agriculture sector or the fishing industry. Others migrate to nearby towns for daily income earning opportunities. The two study sites were chosen to reflect differing contexts to explore whether the social and structural drivers of transactional sex differed in an urban versus rural setting. They were also settings in which we were confident in the availability and effectiveness of referral services to ensure that we could appropriately respond to any referral that may have been needed.

### Sampling and data collection

Sampling for all interviews was purposive. Data were collected through in-depth interviews (IDI) and focus group discussions (FGD) with a variety of community members. As summarised in Table 1, a total of 19 FGDs and 44 IDIs were conducted. In Kampala out-of-school young people 14–17 years of age were sampled through a local NGO’s – the Uganda Youth Development Link (UYDEL) – residential care centre from which young people are provide with educational and vocational training and support. Some women participants aged 18–24 were sampled from beneficiaries of one of UYDEL’s community-based centres. All beneficiaries were made of aware of the study and invited to participate. The individuals who were included in this study were selected based on their willingness to take part, their being in the appropriate age category and their ability to provide independent informed consent. The majority of these participants were young women who had previously been involved in sex work. Recognising the particular contextual realities and unique set of perspectives that this generated, a second FGD was conducted with young women from the community who were aged 18–24 years old but had no involvement with UYDEL or their services. UYDEL also facilitated contact with one mixed-sex secondary school from which young in-school participants were sampled. Students in the desired age bracket were invited to an information session about the study. Those who expressed an interest in participating were given informed consent forms to take to their parents to confirm their consent to participate. From those who returned the form, individuals were selected to reflect a gender-balanced sample of in-school young people aged 14 years and older.

**Table 1:** Participant sampling

Gender and site	Participant age group	KAMPALA	MASAKA	TOTAL
**Focus group discussions**				
Girls and women	14+ in school	1	1	2
	14–17 out of school	1	1	2
	18–24 out of school	2	2	4
	Adult women (35+)	1	1	2
Boys and men	14+ in school	1	1	2
	14–17 out of school	1	1	2
	18–24 out of school	1	1	2
	Adult men (35+)	1	1	2
	Sugar Daddies	–	1	1
**Total**		**9**	**10**	**19**
**Individual interviews**				
Girls and women	14+ in school	2	2	4
	14–17 out of school	2	2	4
	18–24 out of school	4	2	6
	Adult women (35+)	4	4	8
Boys and men	14+ in school	2	2	4
	14–17 out of school	2	2	4
	18–24 out of school	2	2	4
	Adult men (35+)	3	2	5
	Sugar Daddies	3	2	5
**Total**		**24**	**20**	**44**

All other study participants, including adult community members, were sampled through the local government structure. Members of the local council approached individuals in their community whom they knew were in the desired age bracket and made them aware that the study was taking place. Potential participants then met with the field researchers who provided more information about the study and formally invited them to take part. In Masaka, all participants were sampled through the local government structure. A mixed-secondary school was also invited to take part from which in-school young people were sampled in the same way that they were sampled in Kampala. Interviews were gender-matched between the gender of the interviewer(s) and that of the interviewees.

Field researchers were recruited from members of the social science research staff of MRC/UVRI & LSHTM Uganda Research Unit. All field researchers had experience researching sensitive topics including HIV and violence, and of interviewing vulnerable groups including children. In addition to their on-going training on interview techniques, probing and qualitative research procedures, field researches also underwent an intensive one-week training programme on the aims and objectives of this study, the study tools and procedures, the ethical considerations of this study, the informed consenting procedures, and the study’s referral protocol to ensure that any participants who may have required referral support would be appropriately identified and referred. FGDs were single-gender, included an average of nine participants, and were conducted by two field researchers.

Detailed in Additional file 1, FGD participants took part in a vignette discussion. Short stories of adolescent girls under the age of 18 (or which featured a power differential) having sex with men 10 or more years older than them were read and participants were asked to reflect on whether they considered the situation exploitative or not, and why. The scenario was then altered in an iterative manner to explore whether specific changes in the circumstance or age of the characters caused the participants to change their assessment of the situation [41, 42]. The content of these vignettes is summarised in Table 2.

**Table 2:** Summary of sexual exploitation vignettes

Concepts tested	Characters	Vignette
Vulnerability through poverty, and young age	‘Fiona and Peter’	Fiona is from a poor family and is having sex with Peter
		Fiona is 14 and Peter is 25
Inter-generational sex	‘John and Sarah’	Sarah is 17 and John is 45
Power differentials	‘Joan and Andrew’	Joan is in secondary school, Andrew is her teacher
	‘Lucy and James’	Lucy is a live-in domestic worker and James is the married man of the house
“Mutually beneficial” relationships	‘Michelle (poor) and Samuel’	Samuel owns a shop and in return for sex, Michelle receives household supplies like sugar and soap
	‘Michelle (well-off) and Samuel’	Samuel gives Michelle money for mobile phone airtime and to style her hair. He has also given her a mobile phone and promises her a smart phone
Adolescent girls’ and young women’s behaviour	‘Fiona and Peter’	Fiona exchanges sex for alcohol when at Peter’s bar
		Fiona exchanges sex for clothes that Peter trades in town
		Fiona’s parents are strict and restrict her movements. She sneaks out at night and has sex with Peter for transport home

All interviews were conducted in Luganda using a semi-structured topic guide which was jointly translated from English by the study team, all of whom were fluent in English and Luganda. Joint translation was conducted to ensure that all team members had a common understanding of the ambitions of, and nuances in, the language used in the questions and translations. This was also important to ensure that the team were jointly cognisant of any language that could be perceived as stigmatising, given the sensitive topic and potentially vulnerable participants. All interviews were audio recorded. Interviews were conducted in a private location chosen by the study participant(s) in collaboration with the field researchers. Interview locations were selected to ensure that the discussions could not be overheard by others whilst also being safe and conveniently located. FGDs were conducted in a vacant classroom of a school that was not in session. Topic guides were developed based on a review of the literature and on the study objectives and research questions. They were developed through a collaborative process between the study investigators, staff of UYDEL and the research assistants. The final themes that were decided reflected the key themes that were considered necessary to explore the research objectives and questions. Topic guide themes included; friendship, popularity and peer pressure; motivations for, and consequences of, transactional sex; normative views of transactional sex and age-disparate sex; perceptions of young people’s readiness for sex; sexual consent; and views on sexual exploitation.

Efforts to distil the context and extent to which transactional sex was considered exploitative were complicated by the fact that in Luganda there does not exist an exact translation for the word ‘exploitation’. Instead, the concepts of ‘fair/unfair’ and ‘taking advantage’ were used to try to access community members’ perceptions of exploitation or exploitative situations. Synonyms used by participants to describe exploitation included “using”, “misleading”, “force” (which was also used to describe sexual assault) “unfair sex” and “wrong sex”.

### Ethical considerations

Ethical approval for this study was obtained from the Research Ethics Committees of the London School of Hygiene and Tropical Medicine, the Uganda Virus Research Institute and the Uganda National Council for Science and Technology. All adult participants aged 18 years and older provided written informed consent. Those aged 14–17 provided written informed assent. Informed consent/assent was obtained in the same private location that the interview was conducted. It was also emphasised to all participants that their participation was voluntary and that they could choose not to answer any question or to withdraw from the study without giving a reason. They were also assured that their decision not to participate in the study would not affect in any way services that they might have been receiving. UYDEL provided in loco parentis consent for all young people aged 14–17 in their care. In Masaka and for young people who were not under UYDEL care, parents provided written informed consent for their child to participate. Apart from a few men who were identified as sugar daddies, no individuals declined to participate and no parents denied consent for their children to participate. Some in-school students did not return signed parent consent forms and were therefore ineligible.

All FGD and IDI interviews were given a unique identifier number which did not contain any personal data. During the course of the study any handwritten notes were stored in a locked cabinet when not being reviewed by a research team member or before they had been transcribed onto a password-protected computer with any personal identifiers removed. All transcribed interviews were saved onto a password-protected computer without any personal identifiers included. Any data with identifiers, for example the informed consent and assent forms were stored separately from the all other data. Furthermore, all raw data including audio data that had not yet been transcribed or entered into a password-protected computer were located in a secured office and kept in a locked filing cabinet when not on the person of a research team member in the field or while being transcribed.

The study adhered to recommendations under the WHO guidelines for safe and ethical data collection on violence against women [43] and UNICEF’s guidelines on ethical research with children [44]. This included having a referral system in place should any participants be identified to require assistance following their participation in the study.

### Data analysis

Data analysis was guided by a social constructivist theory through which we focused on how individuals created their reality and viewed their world. Our approach was grounded in the work of Berger and Luckman [45] who explored reality creation and the influence of individual meaning based on life experiences, societal and cultural expectations, and rules and norms [46]. We found this theoretical position, in addition to a feminist framing, to be especially useful for exploring emic perspectives of unfair or wrong sex with a variety of participants, particularly when considering the influence of how life experiences, societal and cultural expectations, and rules and norms shape the individual meaning that participants ascribed to various permutations of sex which featured a power differential or was between girls aged below 18 years of age and men aged 10 or more years older. This helped us to access the views and meanings that participants placed on exploitative aspects of transactional sex as well as to explore variations in these meanings and views across participant groups.

Data analysis was thematic and complemented by techniques described in Grounded Theory including deviant case analysis and constant comparison [47]. During regular team debriefings, the data, emerging themes, and potential new lines of enquiry were reflected upon. Emerging findings were also discussed with staff of UYDEL and the wider LINEA team to include their views, experiences and perceptions in the on-going analysis and subsequent data collection. Completed interviews were transcribed verbatim into Luganda and then translated into English [48]. Following intensive reading of the transcripts, a preliminary coding frame was developed by the first author which, supported by NVIVO 10 analysis software [49], was used to code a sample of transcripts. Through on-going comparison of the properties of the data a final coding frame was developed and used to code the remaining transcripts. This included themes that emerged from the data and those identified a priori from the literature and previous research. Separate coding frames were developed for the FGD and IDI interviews and for each of the participant age groups. Data summaries were run using the query function in NVIVO in order to systematically compare the data between sites and participant groups and thus reduce the risk of anecdotal reporting of the data [50, 51]. Through this process, a model of the results was developed as reflected in the findings below in which we note where perspectives varied by age or gender. All names used in the reporting of the results are pseudonyms.

## Results

### Description of the sample

Girls’ and women’s ages ranged from 14 to 47 years. In-school participants were mainly in secondary school with a few in post-secondary, vocational training. The majority of the out-of-school participants had at least some primary-level education. While many out-of-school adolescent girls and young women had a source of income, for most this was insufficient to meet all of their needs. Boys’ and men’s ages ranged from 14 to 56 years. All had at least some primary-level education. Most men aged 25 and above were married and self-employed. Overall, approximately 95% of the participants were Baganda by ethnicity.

### Findings

In general, and across participant groups, transactional sex was condemned on the extent to which it involved sex with a minor or misled a naïve or immature girl; involved lack of consent or the inability to refuse, particularly in relationships characterised by power differentials; worsened the pre-existing status of the girl, or a man’s intention for the relationship was considered inappropriate; the adolescent girl or young woman was considered vulnerable; and the extent to which adolescent girls and young women were considered responsible for ‘their situation’. While many disapproved of transactional sex relationships, this was not always because the relationship was considered inherently exploitative but was instead disapproved of for other reasons elaborated upon below.

#### Sex with a minor or misleading a naïve or immature girl

For most participants, sex with a minor was considered wrong, as a man from Masaka describes:

*“It breaks my heart to find a man as old as I am at 50 with a girl of 16. It is very bad and it greatly impacts on that girl’s life. It is like loading six tonnes on a truck whose maximum recommended load is one tonne” (FGD Adult Men, Masaka).*

Many participants from all age groups also noted that sex with someone below the age of 18 was illegal and could attract punishment:

*“I think that it is proper for a girl to have sex when she has made eighteen years because that girl has reached the decision making age...even with the Ugandan laws, if you have sex with a girl who is below eighteen years, you can be imprisoned” (FGD young women aged 18-24, Kampala).*

Across the data, views were also shaped by the extent to which pressure, deception or manipulation was thought to feature, especially where the adolescent girl or young woman was considered to be young or immature as captured by young men in a FGD in Kampala:

*She is young and needs guidance and he is manipulating her using money and phones. Can’t he find someone who is at least 20 and try manipulating her. She won’t be as easy to deceive as these infants (FGD 18-24-year-old young men, Kampala).*

This pressure was described as considerable, with girls ill equipped to withstand it:

*She can say no but because the man is pressuring her he doesn’t let her say no. When the girl says my parents told me I shouldn’t bring home clothes that they haven’t bought, he says “let me give you money, will they see it too?” When the girl says that my parents will beat me, he says “don’t you have a friend?” He comes up with tricks; “keep this with your friend” it is the man who is wrong. The girl would not have come up with such thoughts* (FGD Adult women, Masaka).

However, a few, particularly male, participants were accepting of a sexual relationship with a minor if she appeared to be physically ready for it:

*I don’t see that as bad because we are told that in African traditional societies, a girl would be married-off by the age of 14 or younger … these days for us to marry, you look at her size and not so much her age. If she is 14 and tall and has good size, then the person who is 25 [can] marry [her]" (FGD in-school boys aged 14+, Kampala).*

Used here, ‘marry’ did not necessarily refer to a formalised legal or cultural union, but instead to having a relationship structured around gendered expectations whereby in return for provision, a man could expect to access sex from a ‘woman’. Many who held this view did not, however, consider adolescent girls’ or young women’s emotional readiness for sexual relationships. Among some participants, particularly women, there was a recognition of a difference between physical and emotionally maturity:

*The difference between being physically mature and emotionally mature is that you can meet someone who is [physically] mature yet they do things like those of a young child [ e.g. play] that means that that person has only grown up in body but the mind is still young (FGD young women aged 18-24, Kampala).*

For many participants across all age groups, age-disparate sex with a minor attracted particular concern. This was explored in a vignette in which ‘Fiona’ was 14 and ‘Peter’ was 25.

“Peter needs to be apprehended and charged with defilement” *(FGD boys aged 14-17, Kampala).*

Many participants condemned his actions with more conviction than if he had been only a few years older than Fiona. As the age of the girl in the vignette was presented as older, for a few, their opposition to age-disparate sex with an adolescent girl waned, even when she was still under the age of consent:

*… God made women in the same way. I am 45 years old but even a 17-year-old girl can have sex with the man that I am sleeping with so if it’s 17 and 45 years then there isn’t much difference (*FGD Adult women, Masaka).

While this view was unusual, it reflected a sentiment that a large difference in age was not always considered exploitative or unacceptable, particularly when the adolescent girl’s body was ‘mature’ or she was already sexually experienced.

#### Lack of consent or inability to refuse particularly in relationships characterised by power differentials

Many participants in all age groups also took issue with sexual relationships that were non-consensual either because they were forced or coerced, or where the adolescent girl or young woman was considered unable to refuse. The inability to refuse was often characterised to occur in situations of asymmetric power where by refusing, the girl risked other negative consequences. Examples of this were where a girl refused the advances of a teacher and consequently risked suffering academically, being otherwise beaten, harassed, or ultimately being forced to leave the school.

*It might be the final year like S4 or S6 [academic years in which national exams are taken] and he asks her to be in a relationship with him. He might threaten her that “if you refuse I will accuse you of an offence and you will be expelled. Remember, it is the final year” (FGD young men aged 18-24, Kampala).*

Many participants recognised a similar inability to refuse for domestic workers whose male employers made sexual advances which they were forced to accept for fear of losing their job, with resulting consequences for them and their dependants.

*He threatens her, that is why whenever he asks her for sex the girl has to agree because he threatens her that once you tell anyone I will chase you away from this home (FGD young women aged 18-24, Kampala).*

This was viewed by some as wrong as it was considered unfair to both the girl and his wife:

*What the boss is doing is not fair; enticing that girl with his money it is not fair to the girl and to the man’s wife (FGD young women aged 18-24, Kampala).*

as well as a violation of the girl’s rights and potentially illegal:

*James is abusing Lucy’s rights as a housemaid. There is a possibility that he even rapes her. If he does not rape her, whatever they are doing is wrong and if he rapes her then he is abusing her rights (FGD 14-17-year-old boys, Kampala)*.

Some participants, however, described circumstances under which sexual relationships characterised by power differentials were not necessarily viewed negatively. In the vignette discussion on a teacher having sex with their secondary school student, some participants, particularly younger and in-school participants, interpreted this relationship as an opportunity for the girl to obtain additional tutoring, money for school fees, snacks, or grades for which she might not have been capable, and thus as an opportunity for future progression. Indeed, many who viewed the situation in this way felt that unfairness arose out of the student being given an advantage over her peers or corrupting them:

*Joan is going to spoil other students. She will tell one of her friends that the teacher is in love with me so the friend will start thinking that I wish that I were in a relationship with a teacher too (FGD young women aged 18–24, Kampala)*

In general, ‘blame’ was apportioned to both the teacher and the girl, though not all recognised the power differential inherent in the relationship and instead blamed adolescent girls for ‘pursuing’ their teachers:

*Such a scenario is not alarming because those things happen – teachers falling in love with students. This is because the students themselves lure the teachers in the way they dress. The teacher is at the blackboard teaching and the girl is there seated badly [with thighs ‘too far’ apart]. Why wouldn’t the teacher go for her? (FGD boys aged 14+ in-school, Kampala).*

In discussing this situation, some men excused teachers’ behaviour arguing that “at times you have to put yourself in his shoes” (in instances where adolescent girls were physically attractive or enticing) and remember that “teachers are also human beings".

*“Your daughter is fed on yoghurt and milk, she looks good, she very enticing. So whenever I look at her while teaching, I get enticed. Remember I am a human being so it is you the parent who caused all this. Because I am a normal man and I notice her beauty” (FGD adult males, Kampala).*

The perception that the adolescent girl or young woman could be disproportionately benefiting was also used by some to evaluate the appropriateness of a domestic worker having sex with her employer:

*The reason why maids get into relationships with their bosses is because they think of where they are coming from and the experiences that they have been through. She compares her home with his home … then starts thinking that if she can get into a relationship with her male boss then her life will improve. That is why maids get into relationships with their male bosses (FGD young women aged 18-24, Masaka).*

Implicit in such narratives was a rationalisation of adolescent girl or young women’s motivation to get into relationships, and a reflection on the benefits derived from such relationships. As such, the power differential did not always feature as a concern, especially where the adolescent girl or young woman was seen to benefit overall.

#### Pre-existing status of the girl and a man’s intention for the relationship

The extent to which transactional sex was condemned also related to girls’ pre-existing status before the relationship. In instances where the girl was perceived to be living a ‘good’ life (for example by being provided for by parents or relatives, being in school, having their needs met and otherwise not struggling in life) or in which her future prospects were promising, a sexual relationship, particularly with the ‘wrong’ man, was at times seen as a risk to this future, and condemned, by a cross-section of participants, on that basis:

*The truth is a teacher having a sexual relationship with a student is very bad, even if they are of the same age...if a teacher decides to relate with a child who is out of school, that is ok...but once a child is in school, it is very bad because they are supposed to be studying, and whenever a child decides to have a relationship with a teacher, he negatively affects her ability to learn (FGD Adult women, Kampala).*

For many participants, however, particularly adolescent girls and young women, adolescent girls were considered to lack reliable opportunities to make a living sufficient to meet their needs and thus sexual relationships with men were often viewed as ‘necessary’ in order to survive:

*I think it is okay for Fiona to have a boyfriend and at that age* [14] *we have to be in school and we have other needs. If Fiona has a boyfriend he will be able to provide for her and may also help her with school fees and she continues her education (FGD in-school girls aged 14+, Kampala).*

In this way, adolescent girls and young women themselves were also seen to use sexual relationships as a means to meet their needs.

Transactional sex relationships were, however, universally condemned as wrong or unfair when men were perceived to have sinister motives for pursuing a girl such as to ruin her life by infecting her with diseases or impregnating her:

*In most cases such men who have interest in young girls have an objective, there is no way that a 30-year-old man can be attracted to a 14-year-old girl. He might have diseases like gonorrhoea which he wants to spread to her so that she also suffers – girls are affected by such diseases more than men (FGD young women aged 18-24, Masaka).*

While a few, mainly male, participants also speculated that some adolescent girls or young women might also be motivated to enter questionable relationships in order to infect their partner with HIV, this was more commonly attributed to men, especially in age-disparate situations, where men refused to use a condom, or where the girl seemingly had little to offer the man apart from sex.

Some participants also noted that a man may be motivated to have sex with an adolescent girl or young woman in order to help her family, although this was often not seen as sufficient to make sex with a minor acceptable:

*[Even] if he is trying to help your family, he should wait for you to grow up instead of defiling you at a young age" (FGD young women aged 18–24 Masaka)*

Some participants, however, described allowances being made for men who were thought to have ‘redeemed’ themselves by ‘doing the right thing’ in situations where their actions should otherwise have attracted consequences:

*A man rented on a certain estate where the landlord had many daughters. He admired one of those girls who was in Primary 5 [official grade age is 10 but students may be older if they have had interruptions to their schooling]. In fact he is the one who broke her virginity. He [the man] asked us for advice but we told him we were going to report him to authorities. Before taking him to the police we spoke to those responsible for the girl. Unfortunately, the girl was an orphan so they [those responsible for the girl] asked the man to take care of the girl until she is ready for marriage. They also asked for the money that they had invested in looking after the girl. The man accepted and up until now the girl is in marriage with the same man (FGD Adult men, Kampala).*

While, as this narrative suggests, this situation was often seen as wrong, the fact that the man ‘did the right thing by the girl’ by marrying her and offering her perceived respectability and financial security, made some participants more accepting of the situation.

#### Adolescent girl or young woman’s position of vulnerability

Vulnerability was often characterised to arise out of individual or household poverty or an adolescent girl or young woman’s desire to complete her education. This vulnerability elicited a lot of mixed responses with relation to the extent to which sexual relationships were seen as exploitative, wrong or unfair. Many, particularly women, often empathised with adolescent girl’s and young women’s situation and described understanding how they might have seen sexual relationships as a means to navigate vulnerability:

*Most of the time … that is the biggest challenge that we have [in our residential zone]. The mother is working, and the father is not working, this greatly affects the mother. So if Fiona gets Peter who is capable of giving her 5000/= ($1.30), remember we only have a single meal, once we eat lunch, then we are done, that affects us, since me as Fiona I would have wanted to eat supper or a sumbusa [samosa] or even the bread that we see at the neighbour’s place. So this 5000/= can do a lot for her. This kind of situation makes it easy for her to get into a relationship with Peter. That is the reason that influences so many young girls like Fiona who is 17 years...this situation where the father is not working and yet her mother is also earning very little (FGD Adult women, Kampala).*

In contrast a few blamed young girls for ‘not being patient’ even in the face of her household’s vulnerability:

*The truth is that what the girl is doing is wrong because she is the first born in a family of five siblings. She is going to plant bad manners among her younger siblings. Showing them that it is right, yet what she is doing [having sex] is not at the right time. Secondly she is fetching more problems for that family … Much as the father is not working, she has to be patient" (FGD Adult men, Kampala).*

Not everyone agreed with this view, however, with many focussing on her young age as evidence of wrongdoing on the man’s part:

*That is child abuse, she if only fifteen years and has not yet got to the age of consent, Peter is only using her, he is defiling her (FGD young women aged 18-24, Kampala).*

Sex with an adolescent girl or young woman, even if she was socioeconomically vulnerable, was not always viewed in a negative light. Indeed for some, particularly young men, the men involved in such relationships deserved ‘credit for helping her’ to meet otherwise unattainable needs.

*You can find that the man wants to pay for her fees and help her in other things. And at 45, the man is mature and knows how to help the girl. So it is okay (FGD in-school boys aged 14+, Kampala).*

As such, the idea that the man was ‘preying’ on, or exploiting a girl’s vulnerability, was contested in the data. This must be understood in the context of structural impediments to adolescent girls’ and young women’s ability to earn an income and the absence of welfare or social security support in the study context. In the absence of state, civil society or familial support, relationships with men were seen by many as a realistic means for girls to meet their needs.

#### Adolescent girls’ and young women’s perceived choice and responsibility for ‘their situation’

Overall, the extent to which a sexual relationship was considered exploitative was also affected by adolescent girls’ and young women’s perceived agency and thus ‘responsibility’ for any negative situations in which they found themselves as a result of their participation in a transactional sex relationship.

***…****they should sit her down and counsel her … because at 14 years she feels that she has to taste everything or it might be the peer pressure at school that is disturbing her so she needs to be beaten and counselled so that she comes back to line. (FGD in-school boys aged 14+,Masaka)*.

Even young adolescents were blamed by some men and women for being in a sexual relationship, and their assumed agency in having entered the relationship, meant that it was not necessarily viewed as exploitative but instead a deliberate means of reaching their objectives. During a vignette discussion with young men in Kampala, the participants added an additional character, Pius, Fiona’s additional, similar-aged boyfriend. By adding him, they were then able to elaborate on their perception that Fiona was using her body to get what she wanted – and could not be argued to have been exploited:

*Fiona is an opportunist she knows she can’t afford to buy the clothes but she can get them using her body. So she gets the clothes in order to look good for Pius [a young boyfriend]. (FGD young men aged 18-24, Kampala).*

This reflected a general sentiment amongst some participants, that some adolescent girls and young women went ‘too far’ in using sex to get what they wanted:

*I think it is not right because at times old men sleep with immature girls who are not yet ready for sex and they end up destroying their reproductive systems. Such girls end up not being able to conceive. I think it is not right and these girls should learn how to set limits* (FGD young men aged 18-24, Kampala).

Here, men were sometimes characterised as ‘hapless victims who were enticed by young women’s sexuality’ and were themselves vulnerable to exploitation:

*When this young girl gets an older man, he is likely to give her everything she wants … because while having sex, the girl can decide to lick the man’s penis just like what they watch on WhatsApp and in the process the man becomes crazy for the girl and the girl gets crazy because of the money (Adult men, Kampala).*

Women were also less sympathetic to the charge of exploitation in situations where adolescent girls and young women were thought to engage in sexual relationships out of perceived greed or instances in which their needs were otherwise provided but they were still discontented:

*If your parents are giving you everything that means that you are still in school. Why should I ask for a phone when I am still in school?* (FGD young women aged 18-24, Masaka).

For a few, such views extended to blaming adolescent girls and young women for any negative consequences that may have arisen because of their relationships, including violence. A group of young men from Kampala elaborated:

*James’ wife might pour acid or hot water or even excreta on her face because she is messing with her husband. Lucy should say no to James because her duty is that of a housemaid (FGD boys aged 14-17 Kampala).*

In a few vignette discussions, participants also speculated that Lucy’s participation in the relationship was tactically intended to enable her to become ‘a second wife’ through entrapping James, for example through pregnancy. Lucy’s decision to participate in a sexual relationship with James was also described to be motivated by how James’ wife treated her:

*If I am Lucy’s boss and I treat her well then she will say no, I will not sleep with her husband but if I am treating her badly then she says that let me do it so that I take over this home, sometimes they even use witchcraft (FGD in-school girls aged 14+, Kampala).*

These views whereby the responsibility of Lucy’s sexual relationship with James lay with Lucy or James’ wife, acted to absolve or attenuate James of his responsibility in the situation, and at times extended to both women and men blaming James’ wife for her perceived failures to “meet her marriage responsibilities”:

*There are some women who leave all the work to their maids, they want the maids to prepare the bath water for their husbands, to lay the beds for their husbands and the maids do this very well. The women are not even always at home and the maids do the work better than the woman of the house that is why the men decide to get into relationships with them (FGD young women aged 18-24, Masaka).*

#### Perceived benefits of transactional sex

Before concluding the findings, it is also important to note that many adolescent girls and young women did not themselves feel that transactional sex relationships were necessarily unfair. Indeed, many emphasised the benefits that they receive in such relationships:

*These days young girls like older men because they have already made their money, when she tells the man that my parents have not given me money for juice, the man will give her 30,000 shillings ($7.86) of which she will spend 15,000 ($3.93) and keep the balance (FGD young owmen aged 18-24, Masaka).*

Some adolescent girls and young women did, however, recognise that while entering into a transactional sex relationship was an agentive choice, once in the relationship, this agency was constrained which they described as a downside to such relationships:

*It is the man [who has power] because he is older than you, he has a lot of experience and he is wiser than you...so if he tells you that we are going to have sex, whether you like it or not you have to accept. Because you accepted to have a relationship with him, when he was making advances at you, you had a right to say no, but since you accepted then you have to dance to his music … accept anything that he wants (18-year-old woman, Kampala).*

## Discussion

While etic definitions of sexual exploitation of children vary, in general, they tend to conceptualise the sexual exploitation of children as sex between a child (under 18) and another person (typically but not always an adult), with unequal power between them, and in which either the young person or a third party is promised, or receives, some benefit in exchange for the sexual activity [1–6]. By these etic definitions, transactional sex is considered sexual exploitation, where the sex involves a minor. Summarised in Table 3, these definitions share some similarities with emic perspectives on wrong or unfair sex. Drawing on data from Central Uganda, the findings of this research highlight, almost universally, that in theory, sex with a minor was considered wrong, with many participants noting that sex with someone below the age of 18 violates the constitution and should, although does not always, attract punishment. As with etic definitions, this reflected concerns about power differentials that put children in a position of vulnerability and inability to refuse sex. This also captures the intersectional disadvantages faced by adolescent girls and young women whereby both their age and their gender, and thus economic and structural disadvantages, place them in a weakened position with relation to sex with older men particularly in the patriarchal society in which this study was conducted.

**Table 3:** Etic definitions versus emic conceptualisations on sexual exploitation

Concept	Aspects of etic definitions of sexual exploitation	Aspects of emic conceptualisations of wrong or unfair sex	Mitigating factors in emic conceptualisations
Sex with a minor	• Child under the age of 18	• Sex with a minor or misleading of a young or immature girl	• AGYW is perceived as seductive or thought to have actively pursued the man and may therefore be considered responsible for ‘their’ situation • AGYW perceived as greedy/discontented with what they have • AGYW benefits substantially or disproportionately • Man’s intentions for the relationship are considered good • AGYW considered physically mature and ‘ready’ for sex
Lack of developmental readiness	• Involvement in sexual activity that a child does not fully comprehend • Child unable to give informed consent • Child not developmentally prepared	...	
Power differential	• Those exploiting the child or young person are able to do so based on power differentials derived from age, gender, intellect, physical strength and/or command of resources.	• Involves lack of consent or inability to refuse, particularly in relationships characterised by power differentials • Child is considered vulnerable or lacking alternative means to meet her needs	
Violates laws and taboos	• Sex violates the laws or social taboos of society	• Sex below 18 violates the law	
Element of exchange	• Situations, contexts and relationships where young people (or a third person or persons) receive ‘something’ (e.g. food, accommodation, drugs, alcohol, cigarettes, affection, gifts, money) as a result of them performing, and/or another, or others performing on them, sexual activities • A child or other person is given or promised money or other form of remuneration, payment or consideration in return for the child engaging in sexual activity, even if the payment/remuneration is not made • Sexual acts are exchanged for goods and services such as housing, food, clothing, drugs or alcohol, protection, better grades in school, or even emotional attention	…	
Harm		• Worsens the pre-existing status of the girl	
Intention		• Man’s intention for the relationship is considered inappropriate	
Benefits	• Recognise that transactional sex may be perceived as beneficial while still being considered exploitative	• Recognise that transactional sex may be perceived and experienced as beneficial	

The findings also illustrate, however, that in practice, sex with a minor in the context of transactional sex was not always considered wrong owing to various mitigating factors. This highlights the point that minor age was not as central to emic conceptualisation of wrong or unfair sex as it is to etic definitions of sexual exploitation. Instead transactional sex was condemned based on the extent to which: it involved sex with a minor or misled a naïve or immature girl; involved lack of consent or the inability to refuse [7, 52], particularly in relationships characterised by power differentials; worsened the pre-existing status of the girl or a man’s intentions for the relationship were considered inappropriate; the adolescent girl or young woman was considered vulnerable, and the extent to which adolescent girls and young women were considered responsible for ‘their situation’, themes that are also reflected in similar work in the region [53].

While etic definitions of sexual exploitation view the exchange elements of transactional sex to be inherently exploitative, emic conceptualisations do not view this transactional element between adults and children as necessarily exploitative. This is in part due to the fact that transactional sex was recognised, particularly by adolescent girls, young women and adult women, as the reality for many adolescent girls and young women who are economically dependent on men owing to structural barriers that limit their ability to meet their needs, especially in increasingly monetised economies [15, 54] (Anonymous (details omited for double-blind review): "That's the price you have to pay for what he has given you". Emic perspectives on social and structural drivers of transactional sex and adolescent girls' vulnerability to HIV in Central Uganda, Under Review). Furthermore, many adolescent girls and young women themselves did not necessarily consider transactional sex relationships to be unfair, even when they were economically or otherwise vulnerable, and entered the relationship in order to try and navigate this vulnerability. Indeed, many adolescent girls and young women felt that they were benefitting from these relationships and were entitled to resources and gifts by virtue of being in a relationship, which reflects how transactional sex is often consistent with long-standing cultural norms of reciprocity [12, 15, 55]. These findings highlight that exploitation cannot be defined in a vacuum. It needs to be interpreted against a backdrop of social norms and structural realities which shape the experience of those involved and how they experience their situation [27, 56–58].

The findings on community perceptions of exploitative, wrong, or unfair aspects of transactional sex described in this paper concord with those of previous studies including the condemnation of adolescent girls’ and young women’s participation in transactional sex relationship out of coercion or force [59], a view that was reflected across all participant groups. Other studies also recognise that for some adolescent girls and young women, participation in transactional sex relationships is driven by vulnerability arising from poverty and absolute deprivation [12, 18, 55]. This characterisation does not, however, provide a full picture for understanding community members’ views of transactional sex. Adult men’s belief that an adolescent girl’s or young woman’s physical maturity signifies readiness for sex has also been found in qualitative research on sexual exploitation in several settings including many in South America [60–62] and highlights how the sexual objectification and pursuit of adolescent girls and young women proceeds in concert with their physical maturation following puberty.

The recognition among young men, adult men and adult women that adolescent girls and young women often have the ability to make apparently agentive decisions to enter into transactional sex relationships is also recognised in the literature. Findings from this study indicate, however, that these relationships, and the girls’ perceived choice to enter into them, was condemned by some participants – particularly by young and adult men [10, 12, 15]. Here participants emphasised adolescent girls’ and young women’s perceived choice in entering the relationship which for some competed with the characterisation that they were exploited. The fact that adolescent girls and young women may be considered to have been exploited even where they have made apparently agentive choices to enter transactional sex relationships has, however, been recognised in the literature and reflects adolescent girls and young women’s constrained power and choices.

Adolescent girls and young women were at times also blamed, mainly by boys and men, for entering such relationships on the basis that they used their sexuality to entice men. In some circumstances it was in fact the man who was seen to be exploited particularly where participants excused men’s behaviour through appealing to an enactment of dominant notions of male sexuality that consider men unble to resist girls and women because ‘they are only human beings’. The apparent choice by adolescent girls and young women in entering transactional sex relationships also resulted in them being blamed by men and a few adult women for any negative consequences that may have arisen from the relationship including HIV, sexual and reproductive health issues, social sanctions, and violence. Blame from both women and men at times also extended to men’s wives whose hypothesised failures to meet their gender obligations was used by both men and women to excuse men’s behaviour. This patriarchal ideology, whereby women are expected to behave properly and if not, negative consequences against them are excused, highlights an important opportunity to address norms that blame women or condone men’s behaviour that results in negative consequences for adolescent girls and young women.

### Strengths and limitations

This study has both strengths and limitations. By including a broad range of participants from two different study sites, the study incorporated the views of an important cross-section of the population. These included sexually active adolescent girls and young women as well as adult men who are in the age group with whom adolescent girls and young women have transactional sex relationships. This provided a unique opportunity to explore understandings of transactional sex as well as the extent and context in which it is considered exploitative. While researchers made considerable effort to build rapport with participants, and were experienced in interviewing on sensitive subjects, we acknowledge that conducting a single interview with participants who may not have been fully trusting of the researchers owing to them being strangers, could have limited the candour with which they spoke. Identifying sexually active 14-year olds was particularly challenging as many may have been shy to discuss their personal experiences, particularly for behaviour that they may have perceived to be stigmatising, especially at a young age. The fact that there does not exist a word in Luganda for exploitation also meant that proxy terms were used to access perceptions of sex that were considered wrong which may not have fully captured the extent to which they were condemned on the basis of being exploitative.

## Conclusion

Etic definitions of child sexual exploitation from multilateral, bilateral, and NGO organisations, and emic conceptualisations of the wrong associated with transactional sex share several key factors including the inappropriateness of sex with a minor. Our paper shows areas of contrast and overlap between emic and etic conceptualisations of exploitation, highlighting views that offer promising opportunities for avenues on which to build through drawing on community-based perspectives. It is rights-promoting to elevate the voices and perspectives of members of communities where transactional sex occurs, rather than blindly applying etic perspectives.

The findings of this study have important implications for understanding how to address harmful or exploitative aspects of transactional sex. We believe that our findings can inform interventions that seek to support adolescent girls and young women to realise their human and reproductive and sexual health rights. As these interventions will be based on an understanding of the perspectives of this target population and their communities, we can expect that they will ultimately be more effective in addressing exploitative aspects of transactional sex than would interventions that do not take emic perspectives into account and thus do not align rights discourses and existing local norms in order to close the gap between them [63].

While the practice is neither seen as inherently exploitative, nor universally wrong, building on existing social norms that condemn sex with a minor, or sex that involves deception, pressure or misleading an immature girl, present an opportunity to mobilise communities to protect adolescent girls and young women at risk. There is also potential to build new social norms that sanction sex with a girl who only ‘agrees’ because she is in a vulnerable position as a means to encourage communities to protect vulnerable adolescent girls and young women. Any intervention must, however, be designed with full cognisance of the social and structural drivers that underlie transactional sex and challenge adolescent girls’ and young women’s ability to provide for themselves without recourse to sexual relationships with men. They must also recognise that girls in transactional sex relationships may not consider themselves as exploited thus requiring engagement with adolescent girls and young women based on their own concerns, aspirations and expectations. As such moving away from conceptualisations of exploitation that position one party as ‘the victim’ and the other as ‘the perpetrator’ may provide space to recognise that benefit may be perceived for both sides, even in exploitative situations. Moving away from a risk-centric conceptualisation of transactional sex may also prove important for ensuring that interventions centralise the views and experiences of adolescent girls and young women so that they resonate with the subjective experiences of those who are involved in the situation. In turn, this may contribute to reducing adolescent girls’ and young women’s vulnerability to HIV infection and other sexual and reproductive health risks.

## Supplementary information

**Additional file 1.** Vignette discussion on sexual exploitation.

## Data Availability

The datasets generated and/or analysed during the current study are not publicly available due lack of ethical clearance to make the data publically available. Data summaries are available from the corresponding author on reasonable request.

## Abbreviations

FGD: Focus Group Discussion
UYDEL: Uganda Youth Development Link
