# Supplementary material for: Community perspectives on the extent to which transactional sex is viewed as sexual exploitation in Central Uganda
Source: BMC Int Health Hum Rights. 2020 May 1;20:11. doi: 10.1186/s12914-020-00228-w (PMC7193338; doi:10.1186/s12914-020-00228-w)
Supplement: Supplementary file 1 — Additional file 1. Vignette discussion on sexual exploitation. [file 12914_2020_228_MOESM1_ESM.docx]

**Appendix 1: Vignette discussion on sexual exploitation**

This afternoon we are going do a story exercise to understand how different individuals feel about young women having boyfriends. But before we start, I would like to ask you a general question.

Are there circumstances where you think it is wrong or unfair for a boy/man to have sex with a girl? Can you think of an example?

Do men ever take advantage of young girls? Can you think of an example?

I would like to read you a short story about someone called Fiona and then ask you a few questions. If you know anyone called Fiona (or any of the other names that you hear) then this is just a coincidence, we just picked the name by chance. As I read it, please think about anyone that you know, or anyone in your community who could be similar to Fiona.

| Fiona who is 17 years old. She is like any other 17-year-old in the community. She is the first born and lives at home with her mother, father, and five brothers and sisters. Her mother sells vegetables at the road side and her father is not currently working.  Fiona is in a relationship with Peter with whom she is having sex. Peter lives in the same community as Fiona.  What do you think/how do you feel about this situation? Is it okay? Is it not okay? Why? | | |
| --- | --- | --- |
| I would like to give you a little more information and then ask you how you feel about the situation | | |
| Age | Of girl | How would you feel if instead of being 17, Fiona was 14 years old while Peter was 25 years old? Would this situation it be okay? Not Okay? Why? |
|  | Of partner | Now imagine that Fiona has a friend called Sarah. Sarah is also 17 and is having sex with a man called John who is 45 years old? What do you think about Sarah’s relationship? Is it okay? Not Okay? Why?  Probe: In your view, is John taking advantage of Sarah? Would you consider the relationship exploitative? |
| Okay, now I would like us to think about some different situations/scenarios | | |
| Identity of partner/power differentials | Teacher | Now imagine that there is a girl called Joan. Joan is in secondary school and Andrew is her teacher at school and they are having a sexual relationship? What do you think about this situation? What could be the benefits? Risks?  Why do you think Joan is in this relationship? For grades? For status? For love? For other benefits? Out of fear?  Why do you think Andrew is in this relationship? For status? For love? For other benefits? |
|  | Employer (house) | Now imagine that the girl is called Lucia and has a job as a house girl in the neighbouring town and that James is married and the man of the house where she works. Sometimes he comes to her room at night and tells her that she must not tell her Madam. After sex he often leaves her with a small bonus.  How do you feel about this? Is it okay? Not okay? Why? What are the risks? Benefits?  Why do you think Lucia is sleeping with James?  Why do you think James is sleeping with Lucie? |
| I would again like us to think about some different situations/scenarios. | | |
| “Benefits” for Michelle | Money | Imagine again that there is a girl called Michelle who is 14. There is also a man called Samuel who owns a shop in town and sometimes gives her gifts or helps her with supplies for home like sugar and soap. In return for his gifts, he expects Michelle to have sex with him.  How do you feel about this situation? Do you think it is fair? Do you think it is Okay? Not okay? Why?  How would you feel if Samuel was a 30-year-old man? |
|  | Consumer goods | Now imagine Michelle and her family are relatively well off, and that at home all the needs are catered for by her parents’ income. Imagine Samuel sometimes gives Michelle money for airtime or to do her hair. One time he even bought her a phone and has promised her he will get her a smart phone.  What do you think about this situation? Is it okay? Not okay? Why?  Why do you think Michelle is sleeping with Samuel?  Why do you think Samuel is sleeping with Michelle? |
| Do you remember Fiona that we spoke about at the beginning? Now let’s think a little more about Fiona as a person. In this case, let’s assume that Fiona is 17 | | |
| Behaviour | Alcohol | When Fiona goes out during the weekend she likes to drink alcohol and often gets drunk,. Peter is the owner of the bar where she likes to hang out with her friends, most of whom are older than her. Peter often gives Fiona drinks for free and then takes her to the back room where they have sex.  What do you think about this situation? Is it okay? Not okay? Why?  Why do you think Fiona has sex with Peter?  Why do you think Peter has sex with Fiona? |
|  | Dress | Fiona likes to follow the latest fashion which mostly involves some revealing clothes. This means that she needs quite a lot of money for her clothes. Peter is 25 and trades clothes in town. If she has sex with him then he will sometimes give her a big discount or even give her some clothes for free.  Is this situation fair? Is it okay? Not okay? Why?  What benefits and risk could there be for Fiona?  What benefits and risks could there be for Peter? |
|  | Hangs out late | Fiona’s parents are strict Christians and don’t like her or her sisters to go to town unless accompanied by them or a relative. Sometimes when they are at all-night prayers Fiona sneaks out of the house to meet Peter and his friends. Peter is 25 and buys her chips, chicken and drinks for the night. They also have sex every time she sneaks out and Peter often refuses to use a condom. One time when she said she didn’t want to have sex with him he said that he would not pay for her to get a taxi or a boda boda home so she ended up having sex with him.  How do you feel about this situation? Is it okay? Not okay? Fair? What are the benefits? Risks?  Why do you think Fiona does this?  Why do you think Peter does this? |

Final question: What would need to change in order to ensure that individuals don’t find themselves in relationships where their partner is taking advantage of them?
